# Report on Asiatic Cholera, as It Appeared in New Albany, Indiana, in the Year 1852

**Published:** 1852-11

**Authors:** Albert De Leszczynski

**Affiliations:** New Albany, Ia.


### WESTERN JOURNAL

OF

MEDICINE AND SURGERY.

NOVEMBER, 1852.

ART. I—*Report on Asiatic Cholera, as it appeared in New Albany Indiana, in the year* 1852. By ALBERT DE LESZCZYNSKI, M. D. of New Albany.

The steamer Midas, from New Orleans for Cincinnati, landed at our port in the night of the 5th of June, having on board, besides her cabin passengers, one hundred and seventy-five deck passengers. The Midas is one of the smaller boats. She had been on her voyage, up to her landing here, about fourteen days. During this time several of her deck passengers had sickened, and sixteen of them, after a short illness, died. On the 6th of June, at 11 o'clock, A. M., I was called upon by a passenger of this boat to see his infant child, which was then in a dying condition. It was attacked by hydrocephalus acutus. Meanwhile, several of our citizens, having heard a report that the cholera had broken out on the Midas, and that several persons had died of it, as also the very deplorable condition of the yet remaining passengers, went on board to assist the needy, and waited upon me to see and assist the afflicted. I went and found the report but too true. The Asiatic Cholera was on board. Two children, a boy and a girl of respectively 11 and 8 years, were dying. Four others, three females and a man, were hurrying towards collapse, and a number of others were suffering with that malign diarrhea usually enumerated among the "premonitory symptoms."

As I afterwards learned, some of our Germano—American citizens—following the dictates of a kind heart, had induced a number of the passengers of the Midas to land and stay here, to avoid further exposure. Four moved into a private family, and twenty-eight of them into a two-story frame house, in the suburbs of our city.

On the 6th of June, at 10 o'clock, P. M., I was called to see the first patients of these immigrants, (of which it was my lot to treat professionally twenty-eight.) One of them was a man, the other a female. Both were attacked with cholera, and both were in collapse. What I understand by cholera, I shall describe under the head of *symptoms*, and only add that the house which had been taken possession of by the immigrants became, in a short lapse of time, the fruitful centre of contagion, whence the epidemic spread in various directions, slaying with unerring hand, all those who did not apply for timely aid. The entire number of cholera patients treated by me, up to the present time, is forty-nine.

*Symptoms.*—I shall here recite all symptoms. To give it a greater order, I shall divide the disease into three stages, (as indeed nature self-divides it,) and delineate the marked symptoms of each division.

Cholera Asiatica observes:

1. The stadium prodromorum—the stage of the forerunners, or that of the premonitory symptoms.

2. Stadium algidum, seu cyanoticum—that of the collapse, and

3. The stadium reactionis—the stage of reaction.

1. STADIUM PRODROMORUM. *Symptoms.*—In some of the epidemics which, at different epochs, have visited large divisions of the earth, premonitory symptoms have been wanting to a great degree; in others they were of longer or shorter duration. *Here*, I observed, objectively and subjectively, the following: Great anxiety; pressure in the præcordial region; vertigo to a greater or lesser extent; slight, or sometimes stronger, headache; ringing in the ears; frequent sighing; dyspnoëa. The appetite is disturbed; the energy depressed; a feeling of weakness, mostly in the lower extremities; and a sensation of a peculiar lightness in the superior posterior part of the thorax. If the patient felt free of headache, the head felt remarkably clear, and the perceptive faculties seemed rather increased. The pulse was quick and easily compressible; the urine became reddish and scanty; the skin, in some instances dry, in others secreted, profusely, a clammy, even cold perspiration. Lastly appeared borborygmi, resembling the moving of gases and fluids, and finally diarrhea. It must not be supposed that all these symptoms took place gradually. Between the first and diarrhea, in some instances, hardly a few hours intervened. Sometimes diarrhea appeared without the slightest admonition. It generally lasted from three to six days but in some cases, hardly as many hours, before changing into the next stage. The diarrhea was not as painful as that accompanying dysentery or typhus; on the contrary, it always, as it were, relieved the patient, who, when it lasted any time, gradually became exhausted. The color of the stools in the commencement, generally, was of a dark green, sometimes light yellow, gradually changing into a lighter one; the quantity copious; the evacuated matter fœtid, but becoming less and less so, as the disease advanced, and when those flocky bodies were in a gradually increasing quantity admixed, which appear in the so-named rice-water discharges. The thirst was great; the tongue white, broad and pale—sometimes livid; the eyes sunk more or less, sometimes surrounded by a dark livid areola; the conjunctiva bulbi, in this stage, remarkably bloodless. Here I must mention the remarkable fact that all, previous to diarrhea, labored under constipation.

2. Stadium Toxicum, Algidum seu cyanoticum.—Among those forty-nine patients treated by me, there was not one where the cyanotic stage had appeared without, although sometimes only a very short, preceding ailment. Of the entire number, twelve cases were collapsed, twenty-six seriously affected, threatening collapsus, and eleven less dangerous. Ten of the collapsed cases complained for several days previous, (and previous to my attendance.) One was collapsed three, another seven hours after the appearance of the first symptoms.

*Symptoms.*—Vomiting and purging of rice-water-like, scentless matters, which continue incessantly, until at last exhausted nature ceases to evacuate. The eyes are sunk into the orbita, the conjuctiva bulbi et palpebrarum is highly injected, the rami of the blood-vessels form a net-work over the eye-ball. The head is hot, covered with a clammy perspiration; the tongue, cheeks, ears, are cold as those of a corpse; the expirations are likewise very cold; respiration very hurried; the pulse, at the commencement of this stage, small and fluttering; soon ceases to beat at the extremities—and even that of the carotids can shortly not be felt. The contractions of the heart are very feeble and intermitting; the respiratory murmur scarcely audible. The thorax is still warm; there is great pain in the epigastrium, like the burning of a coal. The patient complains of violent cramps, which in most cases commenced in the great toe, moving up the legs into the abdomen, when dysponëa takes place. The skin, having lost its turgor, in most cases is covered with cold, clammy perspiration, and corrugated on the extremities and neck, but strongest on the fingers. The thirst is extreme ; the patient continually calls for cold water, which, as soon as taken, is emitted. The papillæ of the tongue are shrunk, that organ self-covered with a slimy, ropy mucus, of the same repugnant smell as that of the slime evacuated by an emetic. The secretion of saliva decreases, and the patients complain of burning in the throat, which partially causes their insatiable thirst. The urinary secretion is entirely suppressed. The patients retained their mental faculties to the last, although some of them, in some cases, were impaired so much, that they seemed to lay in perfect apathy, out of which they could only be aroused by shaking, or very loud talking. This stage lasted with those patients, I observed, from eight to forty-eight hours.

3. Stadium Reactionis.—Although a reaction takes place in the first stage, I shall pass it, and only describe the reaction I observed after collapsus. Only three of my patients died during the latter ; in nine, reaction took place, but only three recovered entirely.

*Symptoms.*—The patients became easier ; the radial pulse returned ; evacuations ceased either entirely, or became less numerous, and strong congestions took place towards head and chest ; the former, during advance of reaction, became hot, the sunken eyes elevated, the face alternately flushed and pale. Perspiration appeared, but the extremities remained cold with those who died, and became warm with those recovering. All lay in a comatose condition ; the eyes were still much injected ; the tongue, when protruded, broad, thick, dry, and trembling. Urine was voided, its color reddish brown, but became soon limpid with those who survived, containing a surplus of area, (as uric acid ) The gums and lips were covered with sordes similar to that in typhus. This period lasted from two to six days, and with one, who recovered, above it.

Therapeutics.—Various were the remedies I employed. The strongest excitantia served in some cases, in others, cerebro-spinantia and astringentia proved themselves of value. In the first stage, plumb. aceticum cum opio; or, if an idiosyncrasy against the latter existed, or as a precaution to children, I gave in its stead hyoscyamus. This, rest, avoidance of cold water and solid food, only permitting rice or barley-water, in very small quantities, soon restored the patients, and the primary changed into a secondary disease only in a few cases. But in every case where this occurred the patient was at fault. During collapse, the strongest stimulants were given. Camphor and aq. ammon. causticæ, diluted with wine, saved one case. I gave equal parts of aq. ammon. and tinct. camphor. ten drops half-hourly, in one ounce of Rhenish wine, and good chicken broth hourly. But, above all, I value the exhibition of Chloroform, from seventy to one hundred drops, in milk or mucilage, repeated every quarter or half hour, until vomiting ceases, or sleep supervenes. This remedy I used only in the latter part of our epidemic, after I had tested its unsurpassed efficacy in a violent case of colica pictonum, which strongly threatened to combine with gastritis, yielding neither to bleeding, opium, alum, croton oil, tobacco, nor cold-water injections—was subdued in a few hours after the subsequent internal exhibition of chloroform.

After the first effects of this substance: cessation of vomiting, of cramps, sleep, and, in some instances, perspiration—opium, gr. $\frac{1}{4}$–$\frac{1}{2}$, with plumb. acetic. grs. ii–iii, and pulv. camphor, gr. $\frac{1}{2}$–i, were of excellent service. These latter remedies I gave until the entire suppression of the alvine evacuations, repeating it every two hours; after which I prescribed quinine sulph., grs. ii–iii, cum opio, gr. $\frac{1}{4}$–$\frac{1}{2}$ pro dosis, repeating it every four or five hours. During the stadium of narcotization—vulgo the typhoid—I gave wine and the mineral acids, of which, particularly acid. sulphur. and alum were of the greatest import.

There were twenty-six cases threatening to run into collapse; chloroform invariably arrested their fatal progress. It proved of the greatest service, when even mosch. oriental. arnica, valerian, and ether, had been hopelessly employed. In all cases of the first and second stage, where the extremities were cold, applications of hot bricks to the feet, bottles filled with hot water surrounding the body, and sinapisms from the chest downwards, were very serviceable. In some cases I employed frictions and fomentations, with hot liquor, as recommended, the blister and the endermic exhibition of morphine on the epigastrium, but with very little success. The use of the lancet was, in every case, even in the stadium reactionis, out of the question. Regarding the local abstraction of blood, I must mention that, although it seemed indicated in one case of reaction, where I applied four cups to the nape of the neck, it proved worse than useless. Emetics of ipecacuanha were, in the first stage, of *some* service. The employment of calomel, either pure or combined with opium, proved always injurious, as also that of mild cathartics in general, even as late as the second day of convalescence, which, in a few cases, even provoked relapse. All gastric symptoms generally subsided after the employment of quinine with opium, as before mentioned; and only when this had been given for perhaps three or four days without producing evacuation, a slight purgative, such as pulv. rhei, grs. viii–x cum hydrarg. mur. m., grs i–iii, could safely be given. All the remedies I have mentioned have received a full and fair trial. Only, those I observed proving injurious, I discontinued immediately, always having before my eyes the wished for end: the saving of human life.

After having stated the remedies which, in my practice, proved *most* successful, we shall pass a small critical review on a few of them, as regards their physiological effects, in order to judge of their respective merits, and the justness of their application.

*Chloroform, Plumbi Acetas.*—Of the other remedies mentioned I shall not treat, as they, at various times, have been fully discussed. Neither mean I to intimate that the combination of acetate of lead and opium has been only used by myself, as I am fully aware that many physicians employ it, and also that the great Dupuytren warmly advocated it.

*Chloroform*—Although its relation to the excitantia is undeniable, it belongs not solely under that head. There can be no doubt, from its external and internal application, that it more justly belongs to the cerebro-spinantia,—of which the narcotic acria also form an order. When applied externally, and undiluted, it causes pain, redness, and blistering, wherefore it must be an acrid. But as it causes disturbances of the cerebrum, as narcosis (anæsthesia) of the motory apparatus, (contractions and contortions of the muscles, especially of those of the face, particularly if given internally,) of the cerebellum and spinal marrow, (convulsions,) it follows that it is a cerebro-spinantium, and as such acts as the most powerful sedative ever known.

When inhaled, or exhibited internally, it at first causes the pulse to increase slightly in frequency, but, presently, as soon as narcotization commences, the circulation becomes slower, the pulse fuller and stronger. Here the stimulating powers cease, and the sedative effects commence. Its actions than extend from the cerebrum to the cerebellum, and elongated marrow to the spinal cord, and necessarily to the peripheric nerves. But, over the great sympathetic nerve and its branches, it has not only no sedative, but a strong stimulating effect, as is thoroughly proved by the violent contractions of the uterus in labor, when this agent is given. Here labor rapidly progresses, although the parturient woman is entirely under its anæsthetic influence.

We shall now pass to review the physiological actions of the *stimulants*, and their changes in the system. I shall not give my individual opinion of processes they undergo and produce, but those of men of acknowledged authority, and add only, that close observation and experiment induce me to adhere to their doctrines.

All stimulants influence, more or less, the functions of the nervous centres,—the substance of the nerves. *Their local actions* are the more intense, the less their volatility and the stronger their affinity to the constituents of the organic structures and fluids. They act upon the mucous membranes and the external integuments, increasing in strength, the finer the epidermis covering them. The primary sensation will be that of heat—even intense burning—and only the most volatile of this class will cause a sensation of cold, a transient decrease of temperature, in consequence of their rapid evaporation. Parallel with the change of the sensible nerves of the dermis, appears an action upon the walls of the blood-vessels and their diameter. They become expanded, and hyperæmia, even congestion takes place in those parts acted upon bythe stimulant. The blood vessels become narrower, the parts acted upon pale, anæmia takes place, which only, after a while, changes into hyperæmia. The local action of stimulants justifies the proposition, that there is a very slender difference between them and the acriä. Stimulants as æther, alcohol, and also chloroform, coagulate protein bodies.

Internally many of the stimulants cause contraction of the muscles of the intestines. Some of them act as mild astringents, and frequently they increase the contractility of the biliary canals, the excretion of bile, and *it seems* as if they had the power to increase the secretion of this matter, by acting chemically upon the blood. All volatile stimulants are rapidly absorbed, and taken up into the circulation, without undergoing peculiar alterations, as other matters will; most so with alcohol, æther, volatile oils, and chloroform. In consequence of this, when taken into circulation they will very rapidly act upon the nervous centres, until acted upon by the oxygen, when they are excreted. Their excretion is nearly as rapid as their absorption. The lungs, kidneys, and perspiratory organs will return that portion of the stimulants which remained unaltered, as proved by the urine, perspiration and exhalation.

Lastly, all stimulants produce a change of function of the *nervous centres* and *circulation.* The former is announced by an exalted condition of the Psyche. Mental depression, sorrow, sleep, apathy, disappear, and give room to a joyful hilarity and contentment. The latter is observable by the stronger contractions of the heart, the fuller, vigorous pulse, and heightened color. Simultaneously we observe an increase of heat. The cause of this I should seek in the abnormally increased supply of carbon, which necessarily asks for an adequate supply of oxygen, which latter is admitted by *accelerated respiration.* The latter and rapid oxydation will elucidate the cause for accelerated circulation and calefaction. How much this, likewise, may influence the nervous centres, and how much their functions, I am not prepared to say.

The ultimate effect of stimulants is a physical and psychical depression. But that this should be only the consequence of the preceding excitement, I must doubt. Should this be so, then excitement would continue as long as the stimulant is retained within circulation. But this is not the case. Although excitement has long given away to depression, the existence of stimuli within the body is proved by their exhalation per lungs. This is the case particularly with æther and alcoholic substances. There must be another cause for depression. According to my view, the blood has undergone a chemical change, a narcotization. This hypothesis is substantiated by the fact that *the fumes of liquor still are exhaled, although a person may have been intoxicated for more than twelve hours!* If all parts of the introduced stimulus had been expelled, how could the fumes still be observed? Here another fact strikes us: the purer the alcoholic substances used have been, the shorter will be narcotization, provided the quantity has been the same.

A short recapitulation and application of the just said, will elucidate the virtues of chloroform. It acts upon the elasticity of the coats of the blood-vessels and capillaries, on account of its energetic *antispasmodic* properties. It increases the mucous membranes, so much so, that even hyperæmia may be caused by it. It coagulates protein bodies, produces contractions of the muscles of the intestines, and promotes the excretion of bile, (as proved by vomiting after the exhibition of a large dose.) What kind of chemical changes it produces upon the blood, and if it really causes increased secretion of bile from it, must yet be decided. Its action upon the nervous centres are well enough known. It exhilerates, soothes and produces a temporary irritable and paralytical torpidity (anæsthesia) of the voluntary apparatus, but an increased action of the ganglionic nerves, and of the organs they enter.

It is best given in milk or mucilages. The dose is from 25 to 75 or 100 drops, repeated every quarter or half hour, as circumstances require. It even has been given with good success in colic to 200 drops pro dosis, and the dose repeated. This I state upon the authority of Dr. Clapp, Sen., of this city, which shows that the evil effects of chloroform are not so great as some physicians maintain. But the exhibition of this, like every other article of the materia medica, is subjected to certain laws, indicated or contra-indicated. It is *indicated* in most spasmodic or neuralgic diseases or conditions of the system, where other remedies may have failed, or where an immediate effect is peremptorily called for. I, on my part, should give it in every case where a safe stimulant and antispasmodic is indicated, and only should find it

*Contraindicated*—1. In a *plethoric subject*, or inflammatory disease, as inflammatory colic, where, previous to its administration a vein is to be opened, when it safely may be given.—2. In a *cachectic subject*, or one laboring under a dyscrasia. In the first case the stimulating powers might be too great, causing apoplexy; in the second, the chemical action of the remedy might decompose the already diseased blood to such a degree that it becomes entirely unfit for nutrition, when death must ensue. We know the baneful influence of alcoholic substances on cachectic individuals. This acts in a similar manner. Although the modus operandi of the substance upon the blood is not perfectly understood, its *effects* we name narcotization.

*Plumbi acetas.*—All soluble preparations and oxydes of lead when brought into the stomach, are acted upon by the secretion of the latter, and intestines, and enter the circulation. They unite with the albumen, but not with the fibrine or hæmatosin of the blood, (Cozzi, 1844.) Lead, when once taken into circulation, acts particularly upon the secretory processes, the membranes and the spinal marrow, with its peripheric continuations and terminations. The motory and trophical nerves are most affected by it. The secretion of saliva, urine, fœcal matters, the perspiration, in short, most all, se.—and excretions are impaired, and only in rare cases we observe salivation, (previous to which, on the gums, the so named

"blue line.") In minute and not too long continued doses, lead will not cause important alterations of function of the nerves, but when continued for a very long period, or when given as oxyde, or salts, in an over dose (ʒss—ʒi or more), or if persons for a long time are exposed to its influence, then the functions of the nerves, ergo their substance is altered (as proved by post mort. sect.) In the stage of consecutive intoxication (as Tanquerel calls it), we observe hyperæsthesis, spasms of diverse peripheric portions of nerves, vertigo, headache, algia, even delirium and coma. This proves the affection of the ganglia, the spinal marrow and brain, the sources of the trophical, sensible, and motory nervous fibres.

When the nervous centres are acted upon by a powerful stimulant as Chloroform, when those alterations ascribed to it have been produced, it becomes necessary to check the profuse secretions, whilst simultaneously we must be thoughtful to stimulate the nervous system to continued action. This will be effected by plumbi acetas with opium. Both are cerebro-spinants, although differing materially in their individual effect. Opium given by itself I found insecure, not so the acetate of lead, which, when given alone, acts as a cerebro-spinal stimulant and astringent. As remarked before, where opium should be contra-indicated, or in children, hyoscyamus will fill its place. Of the other remedies used I shall not speak, as any materia medica will serve this purpose. I should not have spoken so lengthily had not justice to the remedies demanded it. Although perhaps I may be mistaken, I think it wrong in any author who only enumerates his remedies used in a certain disease, without showing his reasons for their administration.

In writing this essay I have not adopted the general rules. My motives in acting contrary to these were, to present to the reader first, the clinical, after which I shall pass to the theoretical part. To comply with this view I will now give the results of (I am sorry to say) the only and rather imperfect *autopsy* I held. The subject of this examination was Henry Schmidt, a German immigrant, aged 39 or 40 years, of robust, plethoric constitution. Autopsy six hours after death.

*Head and spine* I have not examined, as I had neither the necessary instruments with me, nor time to do so.

*Chest.—Trachea and bronchi* slightly injected, of a sticky feel, as indeed were all membranes of the chest; the pleura injected, and the right heart filled with a black, tar-like blood, the former not crepitating when incised. The diaphragm injected.

*Abdomen.*—The liver soft, full of blood of the quality before stated. A *remarkable fact* must be mentioned here. Upon the convex side of the liver, about in the angle formed by two lines, one horizontal, drawn from the incisura interlobularis, continued five inches,—the second, vertical, drawn from the superior posterior margin, continued two and a half inches, I found a *gangrenous patch*, of the size of a ten cent piece; and beside it another of the size of a pea. Depth of gangrenescence, one inch. This corresponded to a pain in the right hypochondrium, complained of by this and other patients. But as I had no other chance of a post mortem examination, I do not know whether gangrene here was *accidental* or *essential*; my belief turns toward the latter conjecture.

*The gall-bladder* was full, containing ℥iv ℥v of dark green bile.

*The stomach*, enormously distended, contained about three quarts, of which two were rice-water-like fluids, containing the specific flocculi, and the third, meat and vegetable substances, which, eaten twenty-four hours previous, although excessive vomiting had taken place, never had been evacuated, nor acted upon by the gastric juice, so that it was easy to distinguish the species. The coats were excessively thin, the mucous membrane in several places *injected.* It was easily scraped off with the handle of the scalpel. *The pancreas* was contracted, anæmic grayish-white, *the spleen* of normal texture and size, filled with tar-like blood; the smaller intestines distended with the same fluid and gas found in the stomach —in several places injected; the large intestines, distended with the same fluid, were otherwise normal. They contained a number of ascarides lumbricoides. I forgot to mention that some of the patients who died evacuated a great number of these worms. *The kidneys* were bloodless, the *bladder* contracted to the size of a large walnut.

ÆTIOLOGY.—Cholera Asiatica, as the name tells, a child of Asia, has been transplanted at various times from its place of birth to Europe, thence to our continent. Its genesis, although enshrouded much in darkness, has, by various observers, been attributed to *malaria.* Although *malaria* is a very vague idea, so much must be certain, that one, causing cholera, must be *sui generis.* Every plant bears its own seeds, and every specific seed generates its specific plant. When the lower division of the organic kingdom follows certain prescribed rules, why, I ask, should not the higher obey like ones? Certain causes produce certain chemical changes in the vegetable kingdom, according to irrevocable laws and specific causes must produce specific results in the animal kingdom, according to like irrevocable laws. Should the highest species of animal, man, only be an exception? As much as his growth, perfection, and decay are carried on by certain rules, so much must be his diseases. As soon as the normal laws conditioning health are infringed by a specific noxiousness entering the organism, so soon will another set of chemical actions be instituted, which, derogatory to growth, perfection, or preservation of the individual organism, will either cause a greater or lesser destruction of the individual fabric—*death or imperfect convalescence*—or the specific noxiousness has been consumed in the process, or annihilated by art, when the primary chemical processes will be supreme again—health. The revelation of the ætiology of physiological and pathological processes, is the aim of the true physician.

If cholera is a specific disease—and no one doubts it—then it must necessarily have a specific cause,—it must be of a contagious nature. This latter has been denied. Every practitioner of the South knows that intermittens perniciosa—vulgo, congestive chills—appears at certain times, endemically and epidemically,—so that the question involuntarily arises in one's mind, can it be contagious? Of the law regulating the genesis of epidemics, endemics, and contagion, anon. Professor Fuchs, of Gœttingen, mentions in his manual of Nosology and Therapeutics, facts which strongly speak for the contagiousness of cholera. Some say it is not contagious, but infectious; some, again, it is portable. What is the difference between *contagium* and *infectio?* Infectio literally means, *the introduction of a mass—of an agent;* whilst *contagium* signifies, *an infection communicated by actual contact*—from *con* and *tango.* The signification of "portable," every one knows, and logic teaches, *when a portable matter can cause a specific disease,* IT MUST BE A CONTAGIUM. I hope that I shall prove that cholera, at least under certain circumstances, can be contagious.

As I mentioned in the introduction, four persons took lodgings in a private family, whilst the other twenty-eight moved into an empty house. H. S., of the first party, died. Over his body I held the post mortem examination. Two men, his host and another, assisted me. Eight days after, his host, Mr. W——t, and myself, and fourteen days after, the other man, were attacked. This man died. He lived about one mile from the scene of action. Nine days after his decease one of his children* died likewise. On the day of H. S.'s death—the first victim—his oldest

---

Caaes marked thus: (*) signifies, not treated by me.

daughter was attacked. The next day a Mr. H——l took her, from charity, into his house, which was situated about one and a half miles from where she had been taken sick. At this time I did not as yet believe in the contagious nature of the disease. Seven days after, Mr. H. was a corpse. A woman, a relative of his, living about one and a half miles from him, in the country, washed the clothing he died in. Eight days after *she* was dead. Five days more, and two children died in the house this wowan had lived in. A neighboring lady, visiting Mr. H., brought the cholera home; she and two more of her family were attacked. Let us return to the first mentioned house of Mr. W——t. His wife was taken ill with it. A brother visiting this family, and residing over one mile from them, brought the disease home. He and five of this family were attacked; the first one of those attacked died. I brought the cholera into my own house. All my colleagues of this place could adduce a number of cases of like import. We are all of the opinion *that cholera can be, and, at all events, this time has been contagious.* When the contagiousness of our epidemic cannot be denied, the question arises: Why has not cholera been contagious in previous epidemics? I only shall speak of what I have seen, not of what I heard. In 1849 I observed this disease in St. Louis and New Orleans. The latter port was the first affected, whence it spread to many landing places of the Mississippi, until arriving at St. Louis. It was in January of that year there appeared the first cases, *imported* by travelers from New Orleans. Cholera was likewise *imported* from Europe—it was not autochthon. Physicians, persons and nurses attending the sick, were attacked, and many even died of its ravages. Does this, too, speak *against* its contagious nature? Some may say that not all persons attending the sick were attacked, and that this fact proved its *non-contagiousness.* But which *acknowledged* contagion can extend its influence over every person?—How many cases of the most malignant form of variola have I attended, and how many victims of this loathsome foe dissected, without being injured by it myself! This proves that cholera, like any other malignant disease, observes the law of "predisposition." Not only this, but other causes may have prevented cholera from becoming in one place more than endemic, or only appear sporadically, although transplanted by direct communication, whilst in the place it emigrated from, it raged as an epidemic. They may be double. To answer the first, I shall quote here part of an article I wrote in 1850.

As nature never transgresses her laws, cholera must have its germ. *I do not doubt but this ens proprium associates with malaria, but doubt that malaria, without the germ of cholera can become cholera proper.* Therefore yellow fever, and all the host of specific diseases malaria is charged with producing, must have their respective germs, which undoubtedly enter into alliance with malaria. Nay, more: I firmly believe that malaria is the nucleus of these different germs; that it only stands in such a relationship to them as the *albumen* to the *vitellum* of the egg. *Ergo, to produce malaria, sulphates are required. Now, if a vicinity or place is not supplied with these bodies, (sulphates of lime and of magnesia,) malaria cannot arise.* Let us suppose malaria and sulphuretted hydrogen the immediate causes of cholera, how could it be possible for these to produce such a diversity of disease? I acknowledge it possible that different degrees, but only of one and the same disease, could be produced, according to the greater or smaller quantity generated. But there are places which contain either more or less of sulphates, and the disease, as an individual hardly varies in intensity. It is possible that when the germ of cholera is introduced, some places are perfectly exempted, whilst others are nearly devastated. Again, at a time of recurrence of the scourge, the first place may have acquired the conditions necessary, whilst the other has not. The second cause is a want of predisposed persons to increase the disease to an epidemic, when the contagion necessarily becomes extinct. Now we inquire about the nature of contagion.—First, what is contagion? This name we apply to all those noxious products generated in the animal organism, which, when transplanted into an organism of as high a standing as that in which this deleterious matter has been produced, will cause absolutely the same organic changes—*disease*—as that which caused its genesis. We shall now pass to the genesis of *epidemics*, from which we will deduce the genesis of *contagion.*

When the atmospherical and telluric noxious influences produce upon the organism so great a chemical change as to cause a disturbance of functions of the body, then we call this abnormal state disease. The abnormal, like the normal state, has its products and educts. When the functions are changed, the organic structure must change likewise; ergo, the products and educts of the body likewise. The more intense the organic and functional changes are, the greater abnormity the products will exhibit. When there occurs one or more cases of the same lesions, the same external symptoms,—also of the same disease, we call them "spontaneous." Should those telluric or noxious influences extend over a larger range of country, causing disease, we say it is endemic; but if they extend over still larger districts, attacking a very great number of individuals, we say the disease has become an epidemic. When during the prevalence of an endemical or epidemical disease, many persons attacked by it are enclosed in relatively small rooms, to which not enough fresh atmospherical air can be admitted, then these rooms will not be filled with carbonic acid, but *with animal products of exhalation*, and perhaps with those of the voided excretions, which will now putrify, and not only produce the original disease, but by far a more virulent one, causing, in some, dyscrasy of the blood, but in all *affecting the nervous centres.* This new product we call *contagium*, and the manner in which it originated its *genesis.* It is not necessary that contagium be generated only in enclosed rooms. If many individuals under similar conditions are attacked in a low malarial region, where change of atmosphere, on account of its geographical position, is impossible or difficult—as in valleys to which the wind has no access—and where, perhaps, yet planetary influences, may exist (as the hot rays of the sun,) there contagion will likewise be generated, and will be the more lasting, as it finds a ready nucleus in malaria. This I conceive to be the process in which the contagium of wide spread epidemics is caused.

In this manner change the following and other sporadic, endemic, and epidemic diseases, into *contagious epidemics,* as yellow fever, scarlatina, variola, the plague, tussis convulsivus, *Cholera Asiatica.* A contagion once generated acts entirely different, on account of its more virulent nature, and when carried to other places, appears there as contagious disease, not passing through the same process described, nor needing those peculiar favorable conditions as are required to its production. It appears as contagium, and will exist as such, as long as circumstances mentioned before do not exclude it. The next question to be answered will be: Of what nature is Cholera Asiatica, or to what genus of disease does it belong? My answer is: *It is an intermittens, holding the same relation to the so-named organic nervous system, as intermittens perniciosa holds to the cerebro-spinal system,—in other words, a primary localization of the typhoid process upon the ganglionic nerves, which becomes transmitted secondarily to the central nervous organs.* L. W. Sachs, of Kœnigsberg, holds it likewise for an intermittens,—but I am not acquainted with his theory. My reasons for the identity of both diseases rest entirely upon physiological and pathological resemblances, as also upon the similarity of localities in which both originate, and the frequent change from one into the other. Let us draw a comparison between them:

### PLACE OF NATIVITY.

| *Intermittens.* | *Cholera.* |
| --- | --- |
| Low marshy grounds on the banks of rivers and swampy places, where malaria can originate. | Low marshy grounds, in the deltoid countries, through which the Ganges passes, and those of Bengal, Ceylon—all malarious districts. |

### CAUSE OF GENESIS:

MALARIA.

### MODE OF ACTION UPON THE SYSTEM:

INTOXICATION OF THE BLOOD.

### CIRCULATION.

| | |
| --- | --- |
| The blood is removed from the peripheric to the central parts. The contractions of the heart are numerically increased. | The blood is removed from the peripheric to the central parts. The contractions of the heart are augmented. |
| During the cold stage, the temperature of the lower extremities is sunken, the skin of the fingers corrugated, lips, and sometimes the extremities of a bluish color. | The temperature of the upper and lower extremities is sunken, except that of the thorax and abdomen.—(Symptoms given before.) |

### NERVOUS SYSTEM.

| | |
| --- | --- |
| The seat of this disease is that part of the peripheric nervous system, which enters into the closest connexion with the vascular. | The seat of this disease are the ganglionic nerves, following primarily their nearest nervous plexuses, and attacking, secondarily, through their spinal fibres, the spinal and cerebral organs. |
| In the ganglia-intermittens we observe a strongly marked cold stage, whilst in the cerebral intermittens it exists only slightly, or not at all. Neither appear in the latter perfect crisis per perspiration and urine. | Here we have the highest expression of the cold stage. When terminating favorably, crisis by skin and urine takes place. |
| As well as cerebral—may change into ganglia—intermittens, the latter may change into the former. A cerebral intermittens may originally attack the trigeminus, from hence passes to the vagus—where it causes disturbance of respiration—following its ramifications until it reaches the ganglionic nerves, and a ganglionic intermittens, may, in a similar but reversed manner, change into a cerebral intermittens. | Cholera commences in the ganglionic nerves, attacks the abdominal plexus, thence follows the relations of the vagus, causing disturbance of respiration, dyspnœa, and finally settles upon the cerebral organs. |
| Intermittens may locate upon the central parts of the abdominal nervous system, when it will cause violent emesis and diarrhea, which is physiologically explained by the intimate relation between the ganglionic system, and the abdominal mucous membranes. | Here we have violent emesis and diarrhea, on account of the localization of the typhoid process upon the ganglionic system, and the relations of the latter to the abdominal mucous membranes. |
| Intermittens is confined to the human species. | Cholera is confined to the human species. |
| This disease, at a time when cholera is prevailing, may change into cholera. It likewise may change into enterotyphus. | Cholera will change into intermittens, and in some instances has changed into enterotyphus. |

NERVOUS SYSTEM—*Continued.*

| *Intermittens.* | *Cholera.* |
|---|---|
| During the cold stage it may end by paralysis, causing hydrops, one kind of which takes place suddenly, by exhaustion of the abdominal nerves. | Cholera in the first stage ends by paralysis. Here accumulation of water takes place, which is evacuated per mouth and anus. |
| In some instances we observe a papular eruption—as auxiliary crisis—in the mouth, nose, the mucous membranes, and even over the entire skin, which by the older physicians has been named "critical itch." | In some instances we meet with an eruption resembling roseola, wherefore it has been named "roseola cholaica," which in every case is a good symptom. I met with it in two cases; both were women. |

I shall add a few physiological facts, as a proof that cholera attacks, primarily, the ganglionic nerves.

*Vagus.*—This nerve—not properly belonging to the ganglionic, on account of its intimate connection with this system, I speak about it here—sends many rami to the lungs and the heart. When irritated, respiration becomes difficult and hurried, and the circulation more rapid. When the contractions of the heart of just killed mammalia have ceased, they may frequently be caused again by pressing the cervical portion of the vagus. E. Weber and Budge have most clearly proved the influence of the vagus upon the heart, by their experiments with the magnetic-electrical rotatory apparatus. When G. Valentin mechanically irritated the cervical portion of the vagus in recently killed dogs, cats, rabits, or horses, he observed distinct motions of the stomach. The latter sometimes became contracted, sometimes forming an incisura from the greater to the lesser curvature, and at other times performing wave-like motions from the cardia towards the pylorus. Anatomy shows that branches of the vagus connect with the solar and hepatic plexuses, which fact elucidates the influence of the cervical portion of this nerve upon the large and small intestines.

*Sympathicus.*—Valentin observed that the cervical portions of the vagus and sympathicus, the inferior cervical ganglion, the rami cardiaci, and cardiac plexus may either produce or alter the contractility of the different portions of the heart. The stomach may be influenced by the sympathicus into four kinds of contractions, one of which is a peristaltic motion of principally the pyloric portion. When Volkman applied the magneto-electro-motor to the thoracical part of the sympathicus proper, violent peristaltic motions of the stomach and intestines ensued. (Experiments made on an asphyxiated cat.) When the same applied the wires to the ramus splanchnicus major, before it enters the cœliac plexus, strong contractions of the stomach were produced. John Muller, Kuerschner, and other physiologists, have observed that a mechanical or chemical irritation of the plexus cœliacus will produce violent peristaltic motions of the smaller intestines. The lumbar and sacral parts of the sympathicus produce strong actions of the larger intestines. M. Sehiff, in his "De vi motoria baseos encephali inquisitiones experimentales," says that when he had extirpated several of the posterior abdominal ganglia of the sympathicus of a frog, he found the peritoneum and the intestines inflitrated, and the latter and the abdominal cavity filled with effused fluids. The present state of our knowledge of the physiology of the nerves is such as to leave plenty of room for inquiry. When, therefore, many points, as regards the nervous pathological influence in cholera, remain shadowed, we have a right to judge by analogy, from the results of experiments instituted upon nerves in their physiological state, on the pathological condition of these identical nerves by the resemblance of their functional disturbances. Footing npon this right, I judge that *Cholera is a primary disease of the ganglia.* When one pathological process—disease—whose nature is disputed, in its symptoms resembles another, of fixed form and undisputed nature, more than any other, we are justified to classify it with the latter process. Upon this ground I classify cholera with *intermittens.* I have forgotten to state, in the proper place, that I observed *one case of cholera change into intermittens perniciosa, and two cases of the latter become cholera.*

Since I wrote this I have read the report of the Committee on Practical Medicine and Epidemics, (of the American Medical Association for 1852,) on which I shall take the liberty to make a few remarks.

As regards the different curative means which proved, in the hands of many, remarkably successful, whilst others complain of the reverse, it is here the place to say that cholera, like every other disease, differs widely in its nature. It may be simple erethic, inflammatory, or torpid. The *erethic form* calls for the least interference—nature needs only to be guided carefully—whilst the *inflammatory form* requires an energetic treatment. Here antiphlogistics, with which men of high authority have had brilliant success, are in their proper place. The third, the *torpid form*, calls for an entirely different course. Here the antiphlogistic regimen would be downright murder. The cerebro-spinants and roborants are in their proper place. The sinking powers must be reanimated, the apathic nerves called into a new action. The difference in the nature of this disease depends not only upon the cholera-virus as regards quantity and quality, but also upon the subject attacked, upon epidemical and terrestrial conditions. The disregard, or the malperception of these important causes, has been the reason for many failures of remedies, which others, at certain times, found unsurpassable. Here I must remark that those remedies which I found beneficial, may be to others, at later times, perfectly useless. Only the nicest discrimination of local and individual constitution, and of that of the then reigning genius epidemicus morborum, will prevent this. The latter must always be considered, as it is of immense import and regulates the selection of our therapeutical agents. All *our* local diseases partake this year, more or less, of the so named "nervous" type.

*Saline treatment.*—Although some hospital reporters may praise it, I do not think the method upon which it is founded a perfectly sound one. The late Prof. Canstatt, of Erlangen, states that experiments made by Dr. Vanderlinden, in the cholera hospital of Brussels, after the method of Stevens, proved not only unsuccessful, but that they seemed to provoke collapsus.

*Emetics.*—In gastric complications, particularly in the erethic form, they are of a salutary effect, but I think not advisable in *every case.* Their chief eclat, after emesis, consists in their general effects. Hufeland says: "These are due to sympathies, depending upon the gastric nerves. The connexion of these nerves with all other parts of the body, gives great importance to these general effects, and is the medium through which vomits affect every system of the economy."

As regards the contagiousness or non-contagiousness of cholera in St. Louis in 1849, I must say that I saw at least *one* physician of the St. Louis Hospital, intimately known to Dr. McP., attacked with premonitory symptoms. This was in January.

My intentions in writing this essay were to give to the profession facts as they appeared here. When I permitted myself to reason, I must be excused, as the import of the principles involved is strong enough to lead astray.—In some instances I have been guilty of repetition, but only where it became necessary to prove or elucidate by it other facts.

New Albany, Ia., Sept. 15, 1852.

